# Association of fall risk factors and non-motor symptoms in patients with early Parkinson’s disease

**DOI:** 10.1038/s41598-021-84720-w

**Published:** 2021-03-04

**Authors:** Kyum-Yil Kwon, Suyeon Park, Eun Ji Lee, Mina Lee, Hyunjin Ju

**Affiliations:** 1 Department of Neurology, Soonchunhyang University Seoul Hospital, Soonchunhyang University School of Medicine, 59 Daesagwan-ro, Yongsan-gu, Seoul, 04401 Republic of Korea; 2 Department of Biostatistics and Data Innovation, Soonchunhyang University Seoul Hospital, Soonchunhyang University School of Medicine, Seoul, Republic of Korea

**Keywords:** Diseases, Medical research, Neurology

## Abstract

The association of non-motor symptoms (NMSs) with fall-related factors in patients with Parkinson’s disease (PD) remains to be further elucidated in the early stages of the disease. Eighty-six patients with less than 5 years of the onset of PD were retrospectively enrolled in the study. We assessed potential fall-related risk factors including (1) a history of falls during the past year (faller versus non-faller), (2) the fear of falling (FoF), and (3) the freezing of gait (FoG). Different types of NMSs were measured using the Montreal Cognitive Assessment (MoCA), the Beck Depression Inventory (BDI), the Beck Anxiety Inventory (BAI), the Parkinson’s disease Fatigue Scale (PFS), and the Scales for Outcomes in Parkinson’s disease—Autonomic dysfunction (SCOPA-AUT). The faller group (37.2%) showed higher scores for BDI, BAI, PFS, and SCOPA-AUT, compared to the non-faller group. From logistic regression analyses, the prior history of falls was related to the gastrointestinal domain of SCOPA-AUT, FoF was associated with BAI, and gastrointestinal and urinary domains of SCOPA-AUT, and FoG was linked to BAI and gastrointestinal domain of SCOPA-AUT. In conclusion, we found that fall-related risk factors in patients with early PD were highly connected with gastrointestinal dysautonomia.

## Introduction

Falling is one of the major determinants of lowering the quality of life in patients with Parkinson’s disease (PD). Falls are common and variably reported to vary between 35 and 90% in patients with PD^1^. It has been widely investigated that falls in patients with PD are associated with many factors including older age, disease duration, severe parkinsonian motor symptoms, in particular axial motor symptom, disabling dyskinesia, cognitive dysfunction, presence of a prior history of falls, fear of falling (FoF), and freezing of gait (FoG)^2–7^. However, risk factors associated with falling in patients, especially with the early stages of PD have been sparsely investigated^8,9^. The previous studies on fall-related factors concerning PD have been performed in the relatively advanced stages of the disease.

Patients with PD might exhibit different types of non-motor symptoms (NMSs) including cognitive impairment, fatigue, anxiety, depression, cardiovascular dysfunction, and gastrointestinal dysfunction. Until now, compared to motor symptoms, NMSs have not yielded uniform results on the association with fall-related factors in patients with PD. Impaired cognition, including frontal executive function or attention, has been occasionally reported to be related to falls in patients with PD^2,3^. On the other hand, some revealed no significant association between falls and NMSs in patients with PD^10^, while others showed that some NMNs including depression, anxiety, and cognitive decline might be related to FoG in PD^11–13^. Therefore, the detailed relationship between various NMSs and falling or fall-related risk factors remain to be further elucidated.

In this study, we sought to investigate the association between fall-related risk factors and various NMSs especially in patients with early PD. After having several meetings for consensus from the literature review^2–9^, we selected representative fall-related risk factors as follows: (1) a history of falls during the past year, (2) FoF, and (3) FoG. Also, various NMSs including depression, anxiety, fatigue, and autonomic dysfunctions were assessed. It is hypothesized that our findings may enable clinicians to figure out the high-risk group for falls with a focus on NMSs in patients with early PD.

## Results

### Comparison of clinical characteristics between fallers and non-fallers with early PD

Out of 86 patients with early PD, 32 patients (37.2%) reported a prior history of falls during the past year. Accordingly, they were categorized into the faller group, while the others were categorized into the non-faller group. The detailed comparison of the clinical characteristics between the faller and non-faller groups is displayed in Table 1. Baseline demographics and motor symptoms were not different between the two groups. However, the daily dopaminergic dosage was higher in the non-faller group (*P* = 0.009) compared to the faller group. For different types of NMSs, the fallers with early PD showed higher scores of BDI (*P* = 0.031), BAI (*P* = 0.040), PFS (*P* = 0.009), total SCOPA-AUT (*p* = 0.003), gastrointestinal domain (GI) of SCOPA (*P* = 0.004), and urinary (UR) domain of SCOPA-AUT (*P* = 0.036), respectively compared to the non-fallers with early PD.

**Table 1:** Demographics and clinical features between fallers and non-fallers with early Parkinson’s disease (PD).

	Fallers with early PD	Non-fallers with early PD	*P* value
	(*n* = 32)	(*n* = 54)	
Age, yr	71.0 ± 10.7	71.2 ± 7.1	0.931
Gender-female	19 (59.3%)	30 (55.6%)	0.729
Disease duration, yr	1.7 ± 1.4	2.0 ± 1.4	0.420
BMI, kg/m^2^	23.9 ± 3.0	23.4 ± 3.5	0.467
Level of education, yr	8.8 ± 5.3	9.5 ± 4.6	0.512
UPDRS-III (motor)	25.0 ± 2.5	22.4 ± 8.9	0.188
HY stage	2.2 ± 0.5	2.1 ± 0.3	0.703
LEDD, mg	60.2 ± 162.5	186.4 ± 234.9	**0.009**
*Non-motor symptoms*			
MoCA-K (global cognition)	20.6 ± 6.5	21.8 ± 4.5	0.370
BDI (depression)	12.4 ± 8.0	9.0 ± 6.4	**0.031**
BAI (anxiety)	8.3 ± 7.4	4.4 ± 4.5	**0.009**
PFS (fatigue)	46.3 ± 14.6	37.2 ± 15.4	**0.009**
*SCOPA-AUT (dysautonomia)*			
Gastrointestinal (GI) domain	5.7 ± 4.0	3.3 ± 2.5	**0.004**
Urinary (UR) domain	8.3 ± 4.7	6.2 ± 4.3	**0.036**
Cardiovascular (CV) domain	1.2 ± 1.8	0.8 ± 1.4	0.272
Thermoregulatory (TR) domain	1.1 ± 1.4	0.6 ± 1.0	0.085
Pupillomotor (PM) domain	0.5 ± 1.3	0.3 ± 1.0	0.336
Sexual (SX) domain	3.0 ± 2.8	4.3 ± 2.2	0.252
Total score	17.5 ± 9.0	12.2 ± 7.1	**0.003**

Data are *n* (%) or mean ± S.D. values.

*PD* Parkinson’s disease, *BMI* body-mass index, *UPDRS-III* Unified Parkinson’s disease rating scale–part 3, *HY* Hoehn and Yahr, *LEDD* levodopa equivalent daily dose, *MoCA-K* Korean version of Montreal Cognitive Assessment, *BDI* Beck depression inventory, *BAI* Beck anxiety inventory, *PFS* Parkinson’s disease fatigue scale, *SCOPA-AUT* Scales for Outcomes in Parkinson’s Disease–Autonomic dysfunction, *GI* gastrointestinal, *UR* urinary, *C* cardiovascular, *TR* thermoregulatory, *PM* pupillomotor, *SX* Sexual.

Boldface indicates *P* < 0.05.

### Logistic regression analysis for a prior history of falls in patients with early PD

**Figure 1:**
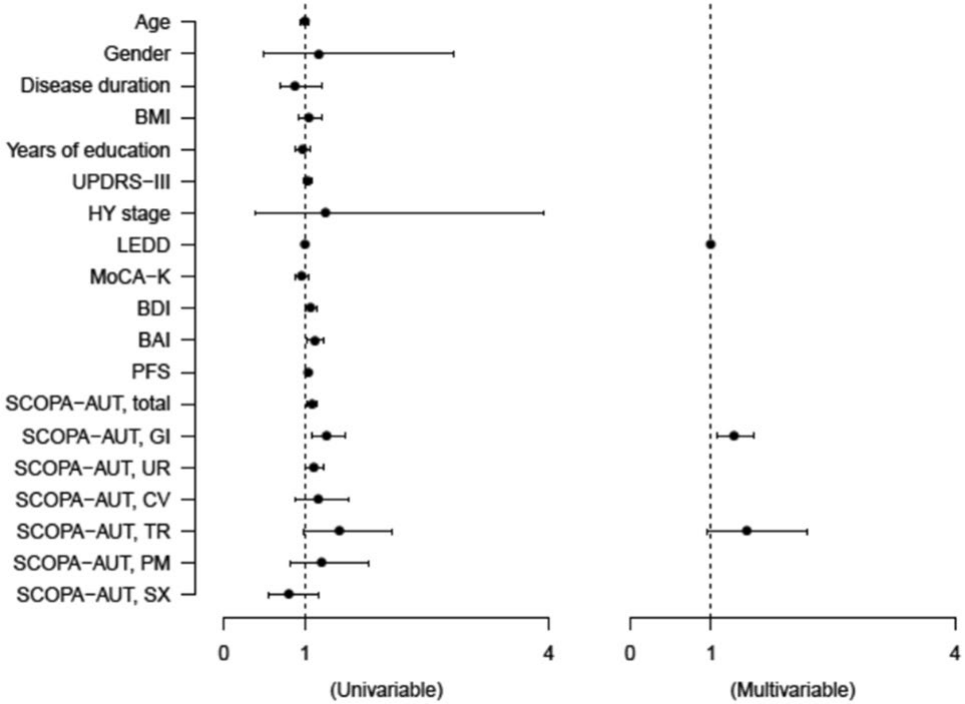
Forest plots showing the logistic regression analyses of clinical characteristics for a prior history of falls in patients with early Parkinson’s disease.

To study the association of NMSs with a prior history of falls in patients with early PD, univariable and multivariable logistic regression analyses were performed, and the results are presented in Fig. 1. In addition, the adjusted odds ratio (adj OR) and 95% confidential interval (95% CI) for the variables were calculated and the outcomes are presented in Supplementary Table 1. It was observed that the previous history of falls was significantly associated with lower doses of dopaminergic medication (adj OR = 0.996, 95% CI = 0.993 – 0.999, *P* = 0.007) and higher score of GI dysautonomia (adj OR = 1.278, 95% CI = 1.078 – 1.514, *P* = 0.005), respectively.

### Logistic regression analysis for fear of falling in patients with early PD

**Figure 2:**
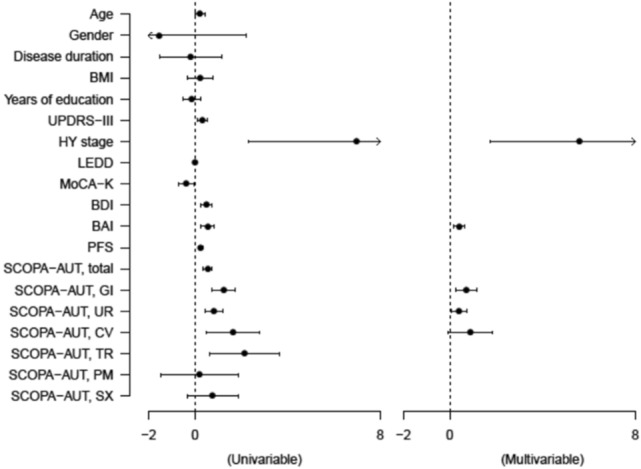
Forest plots showing the linear regression analyses of clinical characteristics for the fear of falling in patients with early Parkinson’s disease.

To identify the risk factors for FoF in patients with early PD, univariable and subsequent multivariable logistic regression analyses were conducted, as shown in Fig. 2. Moreover, the beta (β) coefficients and 95% CI for the variables are presented in Supplementary Table 2. It was identified that FoF was closely linked to higher scores of HY stage (β = 5.591, 95% CI = 1.744 – 9.439, *P* = 0.005), BAI (β = 0.413, 95% CI = 0.167 – 0.659, *P* = 0.001), GI domain of SCOPA-AUT (β = 0.716, 95% CI = 0.242 – 1.191, *P* = 0.004), and UR domain of SCOPA-AUT (β = 0.388, 95% CI = 0.043 – 0.733, *P* = 0.028), respectively.

### Logistic regression analysis for freezing of gait in patients with early PD

**Figure 3:**
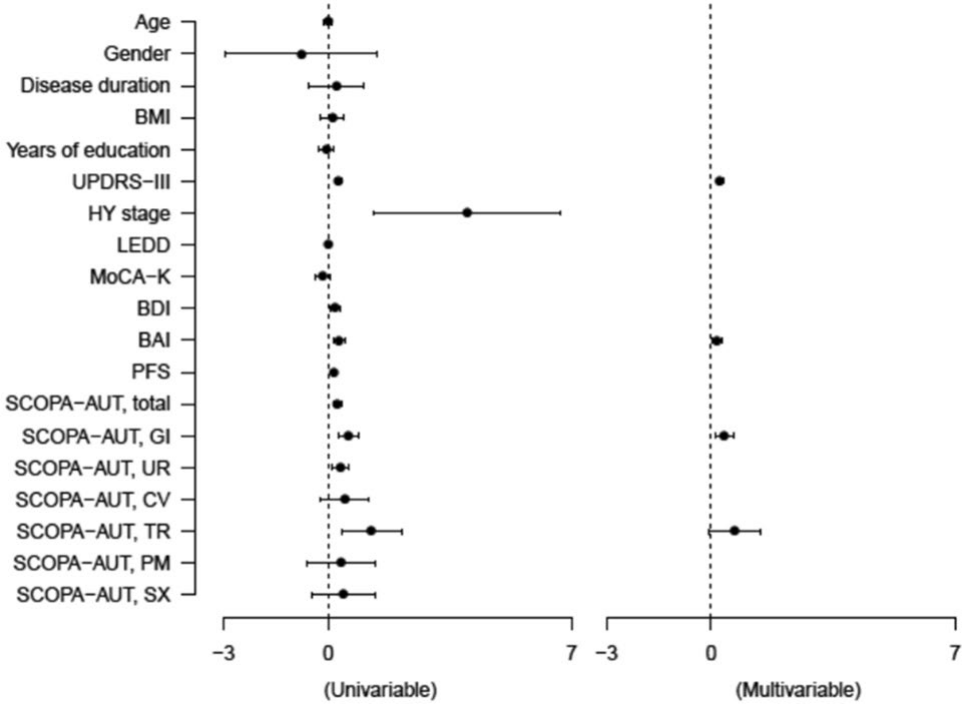
Forest plots showing the linear regression analyses of clinical characteristics for freezing of gait in patients with early Parkinson’s disease.

To uncover the risk factors for FoG in patients with early PD, univariable and multivariable logistic regression analyses were performed as presented in Fig. 3. The β coefficients and 95% CI for the variables are described in Supplementary Table 3. It was identified that FoG was highly connected with higher scores of UPDRS-III (β = 0.244, 95% CI = 0.149 – 0.340, *P* < 0.001), BAI (β = 0.162, 95% CI = 0.013 – 0.312, *P* = 0.034), and GI domain of SCOPA-AUT (β = 0.371, 95% CI = 0.113 – 0.628, *P* = 0.005), respectively.

## Discussion

Previous studies have reported that the prior history of falls ranged between 23 and 46% among patients with early PD^8,9,12^. In line with the literature, the present study showed a similar result that about 37% of patients with early PD experienced falls during the past year. Concerning the association between motor symptoms and falls, neither UPDRS-III nor HY stage was correlated to a prior history of falls in patients with early PD. However, unexpectedly, we found that dopaminergic dose was negatively correlated with the history of falling in patients with early PD. It might be reasonable to infer that proper or enough doses of dopaminergic treatment may prevent or reduce fall events probably by improving motor symptoms in patients with early PD, although UPDRS-III was not different between fallers and non-fallers as per our results.

The pathophysiology of FoG and falls in PD are highly complicated, and the detailed mechanisms have actively been researched. There is evidence that FoG is connected not only with motor networks, but also with cognitive and limbic networks^11,14^. Moreover, a recent study showed that dysfunctional limbic circuitry was significantly associated with FoG in patients with PD, suggesting that the fronto-striato-limbic pathways might be implicated in FoG and falls in PD^15^. Therefore, in line with the literature, our findings may contribute to address the issue of the link between NMSs and fall-related factors in PD.

It has been accepted that anxiety is significantly linked to fall-related factors in the normal elderly^16^. Moreover, very recently, some studies demonstrated the close relationship between fall-related factors and anxiety in patients with PD^12,13^. Likewise, our findings suggest that fall-related risk factors are significantly associated with anxiety, but with neither depression nor fatigue in patients with early PD. Until now, the precise pathophysiologic understanding between falling and anxiety in PD remains unclear. Besides, in the present study, we excluded habitual fallers from the initial stage of patient enrollment because of a potential possibility of atypical parkinsonism, as described previously. Thereby, we supposed that fall-related risk factors may not directly increase anxiety in patients with early PD. One possible explanation is that, unlike depression or fatigue, anxiety is highly associated with dopaminergic depletion in the caudate nucleus^17^, implying that not only falling but also anxiety might be implicated in the striatonigral degeneration of PD.

With regards to the detailed relationship between autonomic dysfunction and fall-related risk factors, autonomic dysfunctions were specifically accessed into 6 domains including GI, UR, cardiovascular, thermoregulatory, pupillomotor, and sexual domain, respectively. Notably, we revealed that fall-related risk factors are strongly connected with GI dysautonomia among 6 domains of dysautonomia in patients with early PD. Our findings suggest that patients with PD and complaints of severe constipation might be at high risk for falls. Accordingly, clinicians need to be concerned when encountering PD patients with GI dysautonomia. Besides, a recent study showed GI dysautonomia was associated with PIGD subtype, compared with tremor-dominant subtype, in patients with early stages of PD^18^. In line with our findings, it implies that early GI and axial symptoms might be interconnected in the pathophysiology of PD. However, to the best of our knowledge, the precise pathomechanism behind the relationship between GI dysautonomia and fall-related risk factors remains unknown. In the future, extensive well-designed studies are needed to address this issue.

The current study has some shortcomings. First, this study was designed as a retrospective study, However, the consecutive patients with early PD were evaluated following a constant protocol and all patients were consecutively followed up periodically in our movement disorder clinics. Second, patients with PD were clinically diagnosed at the early stages of the disease, thereby indicating that some people might have atypical or secondary parkinsonism. Therefore, longitudinal follow-ups of our patients are needed. However, to increase the diagnostic accuracy of PD, we applied more strict criteria including neuroimaging-supported diagnosis, as described previously. Third, we could not cover the whole risk factors associated with falls in this study; we did not evaluate fall-related other risk factors including sleep disturbances such as REM sleep behavior disorder, musculoskeletal problems, visual impairment, vestibular dysfunction, peripheral neuropathy, and detailed medication information. Therefore, a well-designed prospective study is needed to address this issue. Lastly, dysautonomia symptoms were only evaluated based on the survey of SCOPA-AUT. A few objective assessments including orthostatic blood pressure may provide detailed beneficial information to uncover the association between dysautonomia and fall-related risk factors, although SCOPA-AUT is classified as ‘recommended’ for measuring dysautonomia in patients with PD^19^. Especially detailed assessment about GI dysautonomia is warranted for the association of falls.

## Conclusion

Based on our results, we found that that fall-related risk factors were tightly associated with GI dysautonomia in patients with early PD. Clinicians may need to pay attention to falling in the case of PD patients with complaints of constipation, especially in the early stages of the disease.

## Methods

### Participants

This retrospective study was approved by the ethical committee of the Institutional Review Board (IRB No. 2020–03-024) with a waiver of informed consent. We defined the early stages of PD as having disease duration within 5 years from the onset of parkinsonian motor symptoms. Medical records of consecutive patients with early stages of PD, who underwent both brain magnetic resonance imaging (MRI) and dopamine transporter imaging (DAT) imaging for the evaluation of parkinsonism in our movement disorder clinics between July 2017 and February 2020, were carefully reviewed. The initial diagnosis of PD was made according to the UK Brain Bank criteria^20^. For the present study, we only enrolled PD patients showing a considerable response to dopaminergic treatment based on the chart review. During the follow-up periods, we excluded any patient presenting with atypical clinical features including frequent falls, ataxia, abnormal eye movements, or significant cognitive impairment because of a possibility of atypical or secondary parkinsonism. Furthermore, we applied more strict criteria based on the neuroimaging findings; any patient exhibiting atypical findings including hydrocephalus, cerebellar atrophy, or putaminal abnormalities on brain MRI^21^, or displaying the atypical pattern of PD (i.e., unlike a rostrocaudal pattern of striatal dopaminergic loss) on DAT imaging was excluded^22^. Eighty-six patients with early PD were finally recruited in the study.

### Clinical assessments

In our movement disorder clinics, patients presenting with parkinsonism were evaluated with a constant protocol. In addition to demographics, parkinsonian motor symptoms were assessed using the Unified Parkinson’s Disease Rating Scale (UPDRS) part III and the Hoehn and Yahr (HY) stage^23^. Various NMSs in individuals with PD were evaluated using the Korean version of the Montreal Cognitive Assessment (MoCA-K) for global cognition^24^, the Korean version of the Beck Depression Inventory (BDI) for depression^25^, the Korean version of the Beck Anxiety Inventory (BAI) for anxiety^26^, the Parkinson’s Disease Fatigue Scale (PFS) for fatigue^27^, and the Korean version of the Scale for Outcomes in Parkinson's Disease-Autonomic (SCOPA-AUT) for autonomic dysfunction^28^. In addition, patients were interviewed for (1) a history of falls during the past year (classified as faller or non-faller)^5^, (2) the FoF measurement with permission^29^, and (3) the FoG questionnaire with permission^30^.

### Statistics

Student’s t-test for numerical variables or the Chi-squared test for non-numerical variables was used to compare parameters between fallers and non-fallers with early PD. For binary outcomes, univariable and subsequent multivariable logistic regression analyses by backward stepwise selection were conducted. Correlation analyses between the FoF/FoG score and each NMS score were carried out. A *p-*value of < 0.05 was accepted as statistically significant. Statistical analyses were performed by SPSS (version 20.0, IBM Corp., Armonk, NY, USA) and Rex (Version 3.6.3, RexSoft Inc., Seoul, Korea).

### Ethical approval

All procedures performed in studies involving human participants were in accordance with the ethical standards of the institutional and/or national research committee and with the 1964 Helsinki declaration and its later amendments or comparable ethical standards. The ethical committee of the Soonchunhyang University Seoul Hospital approved this study with the waiver of informed consent (IRB No. 2020–03-024).

## Supplementary Information

Supplementary Information
