# Supplementary material for: Association of fall risk factors and non-motor symptoms in patients with early Parkinson’s disease
Source: Sci Rep. 2021 Mar 4;11:5171. doi: 10.1038/s41598-021-84720-w (PMC7933250; doi:10.1038/s41598-021-84720-w)
Supplement: Supplementary file 1 — Supplementary Information [file 41598_2021_84720_MOESM1_ESM.doc]

**Supplementary Tables**

**Association of fall risk factors and non-motor symptoms in patients with early Parkinson’s disease**

Kyum-Yil Kwon^1,*^, Suyeon Park^2^, Eun Ji Lee^1^, Mina Lee^1^, Hyunjin Ju^1^

^1^ Department of Neurology, Soonchunhyang University Seoul Hospital, Soonchunhyang University School of Medicine, Seoul, Republic of Korea

^2^ Department of Biostatistics and Data Innovation, Soonchunhyang University Seoul Hospital, Soonchunhyang University School of Medicine, Seoul, Republic of Korea

Supplementary Table 1. Logistic regression analysis of clinical characteristics for predicting a prior history of falls in patients with early Parkinson’s disease

|  | Univariable | | |  | Multivariable | | | |  |
| --- | --- | --- | --- | --- | --- | --- | --- | --- | --- |
| Variable | Odds ratio | 95% CI | *p* |  | | Odds ratio | 95% CI | *p* | |
| Age | 0.997 | 0.947 – 1.050 | 0.922 |  | | – | – | – | |
| Gender (female/male) | 1.169 | 0.482 – 2.837 | 0.730 |  | | – | – | – | |
| Disease duration | 0.874 | 0.693 – 1.209 | 0.874 |  | | – | – | – | |
| BMI, | 1.051 | 0.920 – 1.201 | 0.463 |  | | – | – | – | |
| Years of education | 0.970 | 0.886 – 1.062 | 0.089 |  | | – | – | – | |
| UPDRS-III | 1.035 | 0.984 – 1.088 | 0.187 |  | | – | – | – | |
| HY stage | 1.254 | 0.399 – 3.944 | 0.690 |  | | – | – | – | |
| LEDD | 0.997 | 0.994 – 0.999 | **0.015** |  | | 0.996 | 0.993 – 0.999 | **0.007** | |
| MoCA-K | 0.959 | 0.884 – 1.041 | 0.320 |  | | – | – | – | |
| BDI | 1.071 | 1.003 – 1.142 | **0.039** |  | | – | – | – | |
| BAI | 1.124 | 1.033 – 1.224 | **0.007** |  | | – | – | – | |
| PFS | 1.041 | 1.009 – 1.074 | **0.011** |  | | – | – | – | |
| SCOPA-AUT, total | 1.087 | 1.025 – 1.152 | **0.005** |  | | – | – | – | |
| SCOPA-AUT, GI | 1.264 | 1.082 – 1.487 | **0.003** |  | | 1.278 | 1.078 – 1.514 | **0.005** | |
| SCOPA-AUT, UR | 1.111 | 1.005 – 1.229 | **0.040** |  | | – | – | – | |
| SCOPA-AUT, CV | 1.167 | 0.885 – 1.539 | 0.273 |  | | – | – | – | |
| SCOPA-AUT, TR | 1.424 | 0.975 – 2.081 | 0.067 |  | | 1.440 | 0.955 – 2.171 | 0.082 | |
| SCOPA-AUT, PM | 1.208 | 0.816 – 1.787 | 0.345 |  | | – | – | – | |
| SCOPA-AUT, SX | 0.803 | 0.556 – 1.159 | 0.240 |  | | – | – | – | |

CI, confidence interval; BMI, body-mass index; UPDRS-III, Unified Parkinson’s disease rating scale - part 3; HY, Hoehn and Yahr; LEDD, levodopa equivalent daily dose; MoCA-K, Korean version of Montreal Cognitive Assessment; BDI, Beck depression inventory; BAI, Beck anxiety inventory; PFS, Parkinson’s disease fatigue scale; SCOPA-AUT, Scales for Outcomes in Parkinson’s Disease - Autonomic dysfunction; Gastrointestinal, GI; Urinary, UR; Cardiovascular, CV; Thermoregulatory, TR; Pupillomotor, PM; Sexual, SX

Boldface indicates *p* < 0.05

Supplementary Table 2. Linear regression analysis of clinical characteristics for fear of falling in patients with early Parkinson’s disease

|  | Univariable | | |  | Multivariable | | | |  |
| --- | --- | --- | --- | --- | --- | --- | --- | --- | --- |
| Variable | $\beta$ | 95% CI | *p - value* |  | | $\beta$ | 95% CI | *p - value* | |
| Age | 0.210 | -0.006 – 0.425 | 0.056 |  | | – | – | – | |
| Gender (female/male) | -1.543 | -5.229 – 2.212 | 0.416 |  | | – | – | – | |
| Disease duration | -0.191 | -1.546 – 1.163 | 0.780 |  | | – | – | – | |
| BMI, | 0.224 | -0.346 – 0.794 | 0.437 |  | | – | – | – | |
| Years of education | -0.152 | -0.535 – 0.232 | 0.434 |  | | – | – | – | |
| UPDRS-III | 0.318 | 0.118 – 0.518 | **0.002** |  | | – | – | – | |
| HY stage | 6.976 | 2.316 – 11.617 | **0.004** |  | | 5.591 | 1.744 – 9.439 | **0.005** | |
| LEDD | -0.005 | -0.014 – 0.003 | 0.215 |  | | – | – | – | |
| MoCA-K | -0.380 | -0.722 – -0.037 | **0.030** |  | | – | – | – | |
| BDI | 0.486 | 0.248 – 0.725 | **< 0.001** |  | | – | – | – | |
| BAI | 0.552 | 0.266 – 0.838 | **< 0.001** |  | | 0.413 | 0.167 – 0.659 | **0.001** | |
| PFS | 0.245 | 0.138 – 0.353 | **< 0.001** |  | | – | – | – | |
| SCOPA-AUT, total | 0.551 | 0.356 – 0.746 | **< 0.001** |  | | – | – | – | |
| SCOPA-AUT, GI | 1.240 | 0.741 – 1.738 | **< 0.001** |  | | 0.716 | 0.242 – 1.191 | **0.004** | |
| SCOPA-AUT, UR | 0.816 | 0.440 – 1.193 | **< 0.001** |  | | 0.388 | 0.043 – 0.733 | **0.028** | |
| SCOPA-AUT, CV | 1.647 | 0.501 – 2.793 | **0.005** |  | | 0.889 | -0.063 – 1.841 | 0.067 | |
| SCOPA-AUT, TR | 2.144 | 0.650 – 3.638 | **0.005** |  | | – | – | – | |
| SCOPA-AUT, PM | 0.196 | -1.489 – 1.881 | 0.818 |  | | – | – | – | |
| SCOPA-AUT, SX | 0.755 | -0.347 – 1.856 | 0.169 |  | | – | – | – | |

CI, confidence interval; BMI, body-mass index; UPDRS-III, Unified Parkinson’s disease rating scale - part 3; HY, Hoehn and Yahr; LEDD, levodopa equivalent daily dose; MoCA-K, Korean version of Montreal Cognitive Assessment; BDI, Beck depression inventory; BAI, Beck anxiety inventory; PFS, Parkinson’s disease fatigue scale; SCOPA-AUT, Scales for Outcomes in Parkinson’s Disease - Autonomic dysfunction; Gastrointestinal, GI; Urinary, UR; Cardiovascular, CV; Thermoregulatory, TR; Pupillomotor, PM; Sexual, SX

Boldface indicates *p* < 0.05

Supplementary Table 3. Linear regression analysis of clinical characteristics for freezing of gait in patients with early Parkinson’s disease

|  | Univariable | | |  | Multivariable | | | |  |
| --- | --- | --- | --- | --- | --- | --- | --- | --- | --- |
| Variable | $\beta$ | 95% CI | *p - value* |  | | $\beta$ | 95% CI | *p - value* | |
| Age | 0.002 | -0.126 – 0.129 | 0.980 |  | | – | – | – | |
| Gender (female/male) | -0.768 | -2.941 – 1.406 | 0.484 |  | | – | – | – | |
| Disease duration | 0.239 | -0.543 – 1.021 | 0.545 |  | | – | – | – | |
| BMI, | 0.127 | -0.203 – 0.457 | 0.447 |  | | – | – | – | |
| Years of education | -0.041 | -0.264 – 0.181 | 0.713 |  | | – | – | – | |
| UPDRS-III | 0.296 | 0.191 – 0.400 | **< 0.001** |  | | 0.244 | 0.149 – 0.340 | **< 0.001** | |
| HY stage | 3.994 | 1.303 – 6.685 | **0.004** |  | | – | – | – | |
| LEDD | -0.004 | -0.008 – 0.001 | 0.157 |  | | – | – | – | |
| MoCA-K | -0.162 | -0.363 – 0.038 | 0.112 |  | | – | – | – | |
| BDI | 0.201 | 0.057 – 0.346 | **0.007** |  | | – | – | – | |
| BAI | 0.315 | 0.149 – 0.480 | **< 0.001** |  | | 0.162 | 0.013 – 0.312 | **0.034** | |
| PFS | 0.157 | 0.097 – 0.218 | **< 0.001** |  | | – | – | – | |
| SCOPA-AUT, total | 0.254 | 0.134 – 0.375 | **< 0.001** |  | | – | – | – | |
| SCOPA-AUT, GI | 0.583 | 0.281 – 0.885 | **< 0.001** |  | | 0.371 | 0.113 – 0.628 | **0.005** | |
| SCOPA-AUT, UR | 0.351 | 0.123 – 0.579 | **0.003** |  | | – | – | – | |
| SCOPA-AUT, CV | 0.479 | -0.207 – 1.165 | 0.169 |  | | – | – | – | |
| SCOPA-AUT, TR | 1.239 | 0.375 – 2.103 | **0.005** |  | | 0.663 | -0.078 – 1.403 | 0.079 | |
| SCOPA-AUT, PM | 0.369 | -0.602 – 1.340 | 0.452 |  | | – | – | – | |
| SCOPA-AUT, SX | 0.433 | -0.485 – 1.352 | 0.337 |  | | – | – | – | |

CI, confidence interval; BMI, body-mass index; UPDRS-III, Unified Parkinson’s disease rating scale - part 3; HY, Hoehn and Yahr; LEDD, levodopa equivalent daily dose; MoCA-K, Korean version of Montreal Cognitive Assessment; BDI, Beck depression inventory; BAI, Beck anxiety inventory; PFS, Parkinson’s disease fatigue scale; SCOPA-AUT, Scales for Outcomes in Parkinson’s Disease - Autonomic dysfunction; Gastrointestinal, GI; Urinary, UR; Cardiovascular, CV; Thermoregulatory, TR; Pupillomotor, PM; Sexual, SX

Boldface indicates *p* < 0.0
